# Editorial: *In vivo* Imaging in Pharmacological Research

**DOI:** 10.3389/fphar.2016.00511

**Published:** 2017-01-03

**Authors:** Nicolau Beckmann, Igor A. Kaltashov, Albert D. Windhorst

**Affiliations:** ^1^Musculoskeletal Diseases Department, Imaging and Histology Group, Novartis Institutes for BioMedical ResearchBasel, Switzerland; ^2^Chemistry Department, University of MassachusettsAmherst, MA, USA; ^3^Department of Radiology and Nuclear Medicine, VU University Medical CenterAmsterdam, Netherlands

**Keywords:** computerized tomography (CT), magnetic resonance imaging (MRI), medical imaging, imaging, pharmacology, positron emission tomography (PET), single photon emission computed tomography (SPECT), ultrasound

The discovery and development of a biologically active molecule with therapeutic properties is an increasingly complex task, highly unpredictable at the early stages and frequently marked, in the end, by high rates of failure. As a consequence, the overall process leading to the production of a successful drug is very long and costly. The improvement of the net outcome in drug discovery and development would require, amongst other important factors, a good understanding of the molecular events that characterize the disease or pathology in order to better identify likely targets of interest, to optimize the interaction of an active agent (a small molecule or a macromolecule of natural or synthetic origin) with those targets, and to facilitate the study of the pharmacokinetics, pharmacodynamics and toxicity of an active agent in both suitable models and human subjects.

This series of articles has been brought together to highlight new developments and applications of imaging techniques with the objective of performing pharmacological studies *in vivo*, in animal models as well as in humans. Imaging has the ability to study various biological and chemical processes non-invasively in living subjects in a longitudinal manner. For this reason, imaging technologies have become an integral part of the drug-discovery and development program and are commonly used in both preclinical and clinical stages. The anatomical, functional, metabolic, and molecular information that becomes accessible through imaging provides invaluable insights into disease mechanisms and mechanisms of drug action.

Computerized tomography (CT) and magnetic resonance imaging (MRI) belong to the so called anatomy-based imaging techniques. They exploit intrinsic tissue characteristics as the source of image contrast. However, both modalities may also rely on the use of agents to highlight some particular contrast. The development of MRI contrast agents has been briefly discussed by Terreno and Aime. The great advantage of CT and MRI in the context of drug research is their translational nature. Thus, they may be used for compound testing in animal models of diseases and further also in clinical studies. Here, Ashton et al. reviewed non-contrast-enhanced and contrast-enhanced micro-CT applications for the study of anatomy and function in small rodents. Recent advances of cardiovascular, neurovascular and renal MRI in small rodents were addressed by Niendorf et al.; Jonckers et al. described the way functional MRI (fMRI) can be used to study the effects of pharmacological modulations on brain function in a non-invasive and longitudinal manner. Finally, Marzola et al. highlighted the use of imaging, especially micro-CT and MRI, for the *in vivo* identification, quantification, and functional characterization of adipose tissues in animal models of obesity, mainly from the point of view of biophysics and physiology.

As the domain of imaging sciences transitions from anatomical/functional to molecular applications, the development of molecular probes becomes crucial for the advancement of the field. Positron emission tomography (PET) and single photon emission computed tomography (SPECT) are molecular imaging techniques of great interest within pharmacological research. They have the ability to provide biomarkers that permit spatial assessment of pathophysiological molecular changes and therefore objectively evaluate and follow up therapeutic responses. They are known primarily for their clinical applications, however, animal studies are also feasible, emphasizing the translational character of these techniques. Here, Declercq et al. illustrated the use of SPECT and PET in the context of drug development for Alzheimer's disease (AD), specifically discussing a number of biomarkers that are supporting emerging clinical therapies for this disease.

Targeted therapy with monoclonal antibodies (mAbs) is an avenue pursued in the context of personalized medicine, particularly for cancer patients. The assessment of *in vivo* biodistribution and tumor targeting of mAbs to predict toxicity and efficacy is an important step toward drug development for individualized treatments. Jauw et al. discussed how PET employing zirconium-89 (^89^Zr)-labeled mAbs, an approach also termed ^89^Zr-immuno-PET, can be used to visualize and quantify the uptake of radiolabeled mAbs in tumors. Overall, ^89^Zr-immuno-PET provides imaging biomarkers to assess target expression as well as tumor targeting of mAbs.

Ultrasound is a classical diagnostic imaging technique often used to locate a source of a disease or to exclude any pathology. It is largely used to visualize internal body structures, such as tendons, muscles, joints or vessels, and internal organs. Besides its ability to provide anatomical information, ultrasound can also display information on blood flow, motion of tissue over time, and tissue stiffness. Compared to other prominent methods of medical imaging, ultrasound has several advantages, including the acquisition of images in real-time, it is portable and can be brought to the bedside, it is substantially lower in cost and does not use harmful ionizing radiation. Drawbacks of ultrasonography include limited field of view, difficulty in imaging structures behind bone and air, and its dependence on skilled operators. Seitz et al. showed that ultrasound is sufficiently reliable to measure acute and chronic changes in the diameter of splanchnic veins in intact rats. Although ultrasound imaging of the abdominal vessels is not novel in experimental research or in the clinics, assessment of diameter changes in multiple splanchnic vessels is new as they relate to venous capacitance.

Recently, ultrasound has also entered the arena of molecular imaging. Paefgen et al. reviewed here the development of bubble-based contrast agents for ultrasound imaging and for imaging drug delivery. The basis for molecular imaging applications is the coupling to the shell of bubbles of specific ligands that bind to marker molecules in the area of interest. Also, bubbles may be loaded with or attached to drugs, peptides or genes. By applying ultrasound pulses, the bubbles are destroyed, leading to a local release of the entrapped agent. The use of microbubble-assisted ultrasound to deliver chemotherapeutic agents has been extensively discussed by Lammertink et al. One specific class of agents that might be of interest for such delivery are S-tanathin functionalized liposomes, as presented here by Fan et al. S-thanatin is a short antimicrobial peptide with selective antibacterial activity (Wu et al., 2010).

Optical imaging adds to the realm of molecular imaging approaches. Main advantages of optical imaging are safety and cost-effectiveness. Major drawbacks, however, are the high scattering and high absorption of light in living tissues. Arranz and Ripoll described the latest advances in optical *in vivo* imaging with pharmacological applications, with special focus on the development of new optical imaging probes in order to overcome the strong absorption introduced by different tissue components, especially hemoglobin, and the development of multimodal imaging systems in order to overcome the resolution limitations imposed by scattering. Despite being mostly limited to small rodents, there is a large interest for optical imaging in the context of pharmacological research, as optical imaging is useful for selecting and validating potential novel probes in an economic and safe (radiation free). The *in vivo* performance of optical probes may predict the outcome of the ensuing and much more involved SPECT/PET tracer development (Sandanaraj et al., 2010).

Animal models have in general been considered of importance in the drug discovery process. On the other hand, the widespread use and evolution of imaging would not have been possible without animal studies. Animal models have allowed, for instance, the technical development of different imaging tools and probes. Santos et al. have critically discussed the value of animal models in the context of cardiovascular imaging.

The focus in this series has been dedicated to *in vivo* macroscopic imaging applications within pharmacological research. Nonetheless, microscopic imaging has also an important role to play in this domain. As an example, Xu highlighted the latest advances in hepatotoxicity, cardiotoxicity, and genetic toxicity tests utilizing cellular imaging as a screening strategy.

In the domain of drug discovery, the pharmacological and biomedical questions constitute the center of attention. In this sense, it is fundamental to keep in mind the strengths and limitations of each analytical or imaging technique. In this series, our aim was to illustrate the fact that the judicious application of a given technique to search for answers to manifold questions arising during a long and painstaking path will continue to rely on imaging as a must-have tool in drug discovery and development.

## Author contributions

All three authors helped organizing the research topic, inviting authors, reviewing manuscripts and writing the editorial.

### Conflict of interest statement

NB is employed by Novartis Pharma AG, Basel, Switzerland. The other authors declare that the research was conducted in the absence of any commercial or financial relationships that could be construed as a potential conflict of interest.
